# A retrospective case series of Fournier’s gangrene: necrotizing fasciitis in perineum and perianal region

**DOI:** 10.1186/s12893-020-00916-3

**Published:** 2020-10-30

**Authors:** Nan Zhang, Xin Yu, Kai Zhang, Tongjun Liu

**Affiliations:** 1 General Surgery, Jilin University Second Hospital, No 218 ZiQiang Street, Changchun, 130000 China; 2 Department of Pediatrics, Jilin University First Hospital, No 71 Xinmin Street, Changchun, China

**Keywords:** Fournier’s gangrene, Necrotizing fasciitis, Infection

## Abstract

**Background:**

To describe the clinical characteristics and management for Fournier’s gangrene. Experience summary and literature references are provided for future treatment improvement.

**Methods:**

We retrospectively reviewed the cases diagnosed with Fournier’s gangrene in our department from June 2016 to June 2019. Clinical data, including manifestation, diagnosis, treatment and outcomes for Fournier’s gangrene were presented.

**Results:**

There were 12 patients enrolled in this paper, with the average age of 60 years old. It showed a male predominance with male-to-female ratio of 6:1. The average of laboratory risk indicator for necrotizing fasciitis (LRINEC) score was 10.1. Diabetes mellitus was the main predisposing disease. 11 patients received emergency debridement and 1 patient died of sepsis on the 2nd day after admission. The mortality rate was 8.3%. 6 cases developed complications, including sepsis, pneumonia, renal and heart failure. Negative pressure wound therapy (NPWT) was applied in 10 cases, while the rest 1 received normal daily dressing changes because of fecal contamination. Flaps were utilized in 2 patients to cover the defect, including one with advancement flap and one with pudendal-thigh flap, while others received secondary suture, secondary healing, skin graft or combined management. No relapse was observed during the follow-up visits.

**Conclusions:**

Fournier’s gangrene is a life-threatening infection that requires early diagnosis and surgery intervention. The predisposing disease, clinical manifestation and LRINEC score should be taken into comprehensive consideration, which is helpful for timely diagnosis. Moreover, further successful treatment depends on the aggressive debridement, broad-spectrum antibiotics therapy, wound management and closure choice.

## Background

Fournier’s gangrene (FG) is a special type of necrotizing fasciitis derived from the skin and subcutaneous tissue infection in perineum or perianal region. The first formal description of FG can be traced back to 1883, and FG is characterized by its rapid onset in males, fast progression, and inconspicuous cause. Nowadays, FG has been recognized as a kind of life-threatening disease with high mortality. According to the existing reports, the mortality of FG can reach up to 29.6% [1] and rise to 35.7% [2] due to delayed diagnosis and insufficient treatment. FG shows a male dominance, and its incidence among the US residents is estimated to be 1.6/100,000 males, which accounts for 0.02–0.09% of total admissions [3]. Besides, FG tends to occur in patients over 50 years, or complicated with diabetics, obesity, malignant tumor and immune disorder. With the increasing populations of these predisposing factors, the incidence of FG also rises accordingly. However, the incidence is still considered to be underestimated because of the insufficient recognition by clinicians. Due to the specific location, FG is usually accompanied by mixed bacteria infections. The infection spreads along the deep fascia, and the lower abdomen or thigh can also be involved as the disease progresses. Most patients exhibit systemic infection symptoms, such as high fever and chills, while some may develop septic shock or organ failure.

At present, the treatment for FG includes fluid resuscitation, nutrition support, surgical debridement, wound management and closure. The clinicians are still encountered with great challenges in the treatment for FG, even though a long time has passed with development in antibiotics therapy and medical care. This study aimed to retrospectively analyze patients diagnosed with FG, meanwhile, the demographic data, laboratory results, clinical management and outcomes of these patients were also presented to provide references for treatment improvement.

## Methods

Patients diagnosed with FG between June 2016 and June 2019 were enrolled in this study. The clinical data of patients, including demographic characteristics, wound manifestation, biochemical criterion, and management protocols, were also recorded. Herein, FG was diagnosed according to the following points: (1) Characteristic signs of infection, such as fever, gentleness, swelling, erythema with vague boundaries, subcutaneous crepitus (gas on ultrasound), excessive pain, and the expansion of infected area at a maximum pace of 2–3 cm/h. (2) Systemic toxicity signs, for example, high white blood cell (WBC) level (> 15.0 × 10^9^/L), metabolic acidosis with elevated lactate level (> 2 mmol/L), a significant decrease in platelet level (< 100 × 10^9^/L), disseminated intravascular coagulation (DIC) or multiple organ failure (MOF) at the end-stage. (3) Laboratory risk indicator for necrotizing fasciitis (LRINEC) score ≥ 8 [4]. (4) Full-thickness skin necrosis, vascular thrombosis and infection spreading along the deep fascial plane discovered intraoperatively. In this study, patients who abandoned the treatment and were lost to follow-up were excluded.

**Fig. 1:**
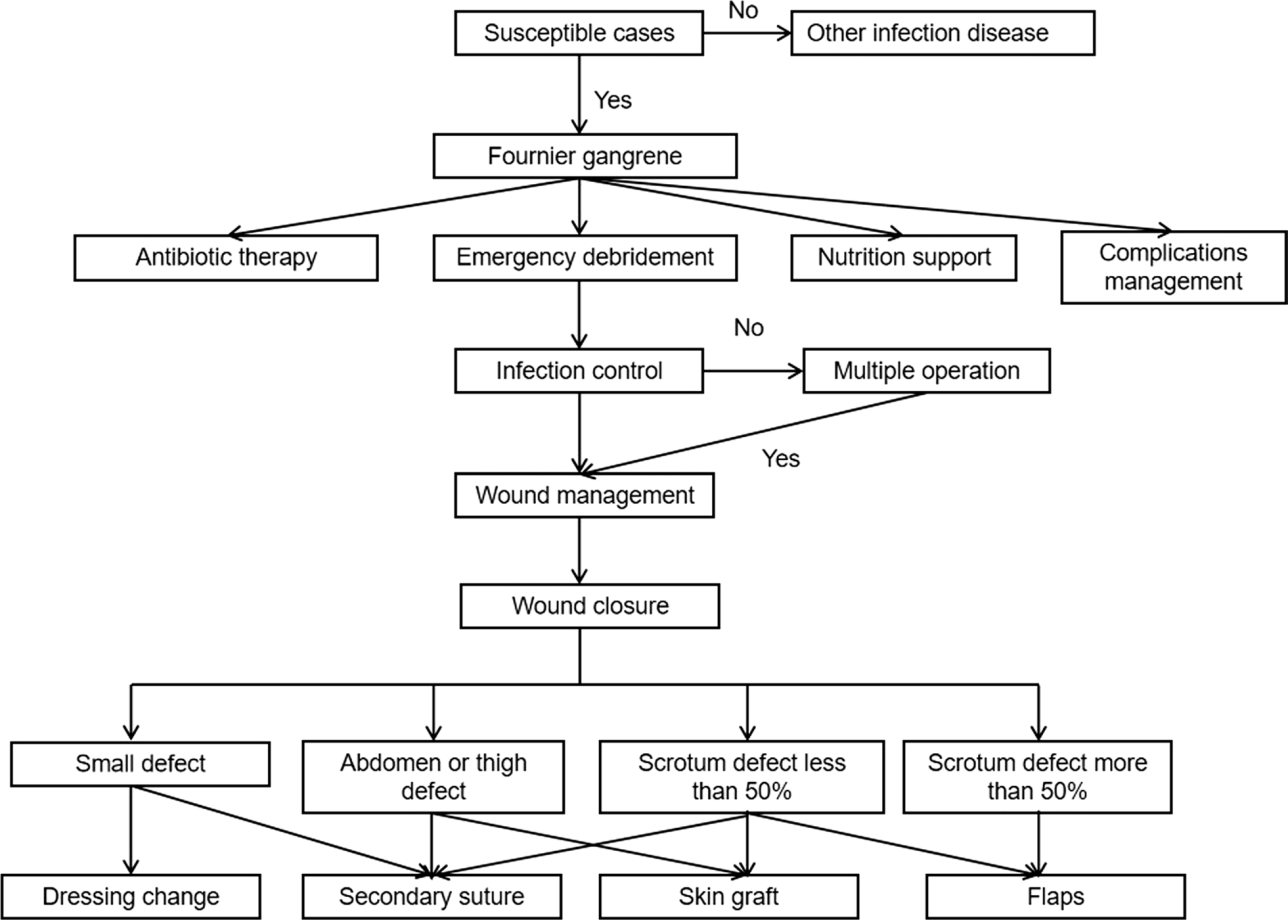
Treatment protocols

The treatment protocols are presented in Fig. 1. Upon admission, the susceptible cases received emergency debridement. The definite diagnosis was made intraoperatively. As for antibiotics therapy, the third-generation cephalosporins combined with ornidazole were used to cover both the Gram-negative aerobic and anaerobic bacteria. For severe cases, meropenem or vancomycin was given depending on the results of bacterial culture. Other treatments included nutrition support, blood transfusion and ion disorder treatment. Repeated debridement was performed in the following days. Complications, including pneumonia or respiratory failure, were treated with ventilator therapy, while continuous renal replacement therapy (CRRT) was adopted for cases with renal failure.

When infection was under control, negative pressure wound therapy (NPWT) was fixed with a pressure ranging from − 120 to − 125 mmHg, and the device was changed every 3–7 days. The duration of changing NPWT depended on the firmness of the fixation dressings. NPWT was abandoned only when the dressings often leaked due to fecal contamination. For patients with ostomy, they were in the care of professional ostomy therapists. In the absence of obvious necrotic tissue or the presence of red-pink new granulation tissue, the subsequent wound closure was considered. For small defects, suture, advancement flap or conservative dressing change was adopted for wound healing. Concerning the larger defect in the abdomen or thigh, the wound was closed by skin graft. For scrotal defects larger than 50%, the pudendal-thigh flap was used.

The study protocol was approved by the Ethics Committee of Jilin University. All data used in this study were collected by a principal doctor. The personal information of patients was protected following the Chinese law, and only the case numbers were provided for identification. The Statistical Package for the Social Sciences 25.0 (SPSS, IBM Crop, Armonk, NY) was employed for statistical analysis. Categorical and continuous variables were expressed as percentages or frequencies and mean, respectively.

## Results

The details of the enrolled patients are presented in Table 1. As observed from the table, altogether 12 patients diagnosed with FG were enrolled in this study, with the average age of 60.0 (range, 45–71) years old, and the male-to-female ratio of 6:1. In terms of the predisposing diseases, diabetes mellitus (DM, n = 8, 66.7%) ranked the top place, followed by abscess (n = 5, 41.7%) and fistula (n = 3, 25.0%). Other predisposing diseases included renal failure (n = 2, 16.7%), leukemia (n = 1, 8.3%) and trauma (n = 1, 8.3%). Moreover, 8 patients (66.7%) had more than one predisposing disease. 6 cases developed complications, including sepsis (n = 3, 25.0%), pneumonia (n = 2, 16.7%), renal failure (n = 1, 8.3%) and heart failure (n = 1, 8.3%). Additionally, the bacteria cultured from the wound included *Escherichia coli* (n = 5, 41.7%), *Enterococcus faecium* (n = 4, 33.3%), *Staphylococcus aureus* (n = 2, 16.7%), *Pseudomonas aeruginosa* (n = 2, 16.7%), Acinetobacter (n = 1, 8.3%) and methicillin-resistant *Staphylococcus aureus* (MRSA, n = 1, 8.3%); meanwhile, 3 patients had multiple mixed bacterial infections. The average LRINEC score was 10.1 (range, 8–13). As for fecal diversion, 3 patients received colostomy and 1 underwent cystostomy. Only one patient died of sepsis on the 2nd day after admission, yielding the mortality rate of 8.3%. In the process of wound management, NPWT was applied in 10 cases, while the rest 1 received normal daily dressing changes because of fecal contamination. With regard to wound closure, flaps were utilized in 2 patients to cover the defect, including one with advancement flap and one with pudendal-thigh flap, while others received secondary suture, secondary healing, skin graft or combined management. The flap and skin graft were performed by burns or plastic surgeons. No relapse was observed during the follow-up visits.

**Table 1:** Clinical characteristics of the 12 enrolled patients

Patient ID	Demographic characteristics			Clinical manifestation			LRINEC score	Faecal diversion	Wound management	Closure method
	Age	Sex	Predisposing disease	Location	Complications	Bacteria culture				
1	66	M	Diabetes mellitus + fistula	Scrotum	None	*P. aeruginosa*	9	None	NPWT	Skin graft
2	48	M	Renal failure + abscess	Crissum + scrotum	Pneumonia	*E. coli*	11	Colostomy	NPWT	Secondary healing + suture
3	62	F	Diabetes mellitus + abscess	Perineum + crissum	None	*E. faecium*	8	None	Dressing changes	Secondary healing + suture
4	64	M	Diabetes mellitus + fistula	Scrotum + penis	Pneumonia	*E. coli* + *E. faecium*	10	None	NPWT	Secondary suture + skin graft
5	60	M	Renal failure	Scrotum	None	*S. aureus*	10	None	NPWT	Flaps
6^#^	71	M	Diabetes mellitus + fistula	Scrotum + thigh + penis	Sepsis	*E. faecium*	13	None	–	–
7	59	M	Diabetes mellitus + leukemia	Scrotum + thigh + abdomen + penis	Renal failure + sepsis	*E. faecium* + MRSA	9	Colostomy + cystostomy	NPWT	Flaps + Secondary suture + skin graft
8	45	M	Trauma	Scrotum + penis	None	*S. aureus*	8	None	NPWT	Secondary suture
9	60	M	Abscess	Crissum + thigh	None	*E. coli*	9	Colostomy	NPWT	Secondary healing + suture + skin graft
10	62	M	Diabetes mellitus + abscess	Scrotum + crissum	Heart failure	Acinetobacter	11	None	NPWT	Flaps + secondary healing
11	57	M	Diabetes mellitus	Scrotum + penis	None	*E. coli* + P. aeruginosa	10	None	NPWT	Skin graft
12	66	F	Diabetes mellitus + abscess	Perineum + abdomen + buttock	Sepsis	*E. coli*	13	None	NPWT	Secondary suture + skin graft

6# patient was dead due to sepsis

## Case presentation

**Fig. 2:**
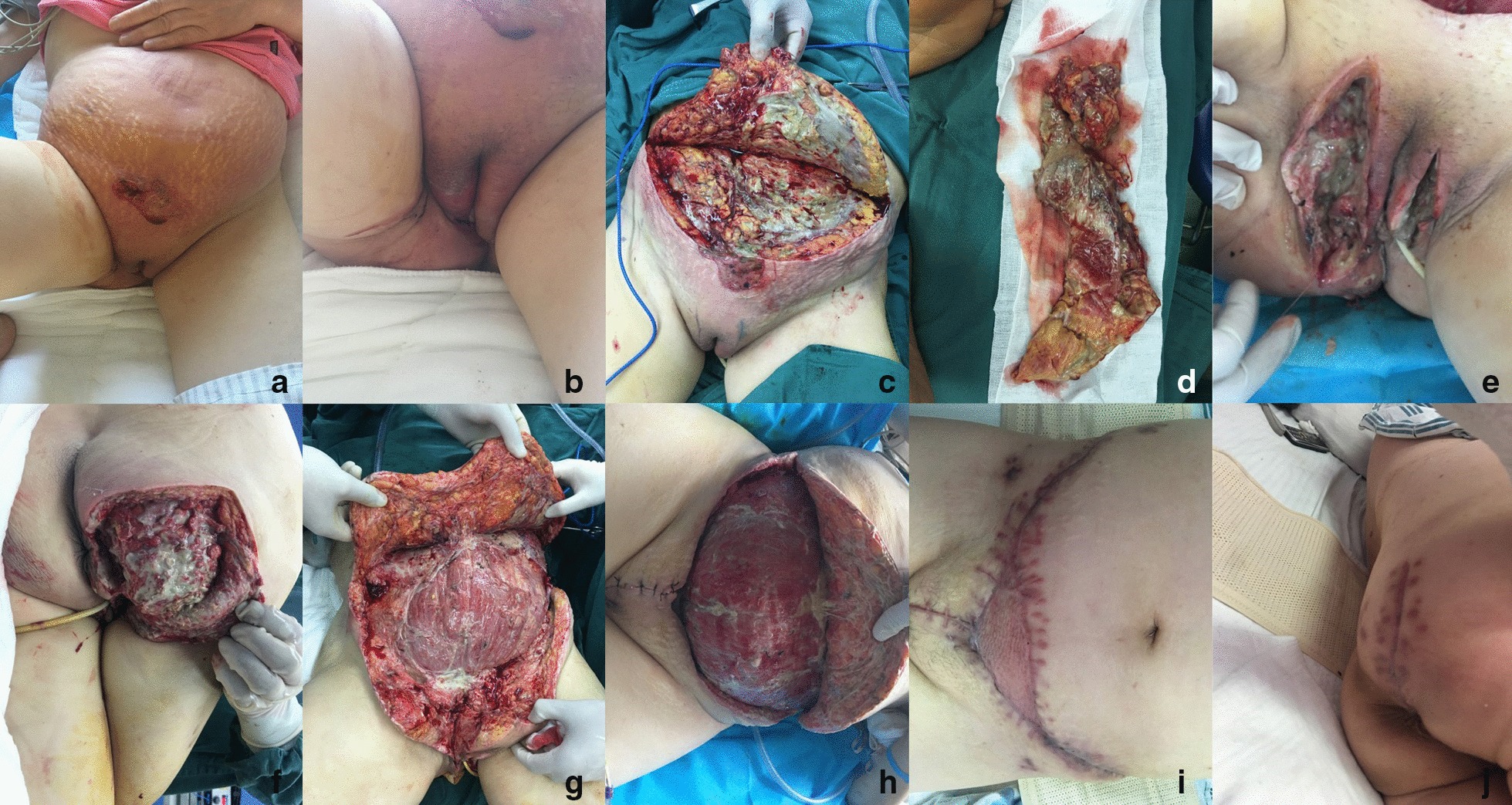
**a** Clinical manifestation after admission in abdomen view; **b** clinical manifestation in perineum view; **c** emergency surgical debridement was performed to control the invasive infection that involved the subcutaneous tissue and fascia, but muscle involvement was not obvious; **d** the excised necrotic tissue; **e** wound in perineum; **f** wound in right buttock; **g** repeated debridement was performed on the 5th day after admission; **h** wound bed manifestation after NPWT; **i** abdominal wound was closed by secondary suture and skin graft; **j** wound in right buttock was closed by secondary suture

A 66-year-old woman was admitted into our department complaining about perineal abscess for 2 days (No 12 in Table 1). Skin necrosis was seen in abdomen and perineum. The swelling and inflammation involved abdomen and perineum (Fig. 2a, b), with a tendency of progressing to right buttock. Crepitus was heard in the abdomen. The body temperature was 38.3 ℃, the blood pressure (BP) was 88/54 mmHg, the heart rate (HR) was 103 beats/min. In addition, the lactate level was 2.9 mmol/L, and WBC was 19.0 × 10^9^/L. In terms of treatment, Ceftriaxone (2 g/time, 1 time/day, Shanghai Roche Pharmaceuticals Co., Ltd) and ornidazole (0.5 g/time, 2 times/day, Sichuan Kelun Pharmaceuticals Co., Ltd) were applied empirically. An emergency operation was performed at 6 h after admission under general anesthesia. Intraoperatively, a widespread infection progressing along the deep fascial plane was observed, which showed the manifestations of tawny pus, putrid odor, vascular thrombosis and skin necrosis (Fig. 2c–e). Bacteria culture revealed the presence of *Escherichia coli* infection in both the wound bed and blood, and the original empirical antibiotic treatment regimen was maintained. At the same time, an insulin pump was applied to control the blood glucose at about 8–10 mmol/L. Emergency operation was carried out to control the infection, while the other two operations were performed on the following 5th and 11th days to remove the residual necrotic tissue and to culture the wound bed (Fig. 2f, g). Other treatments included parenteral nutrition support (25–30 kcal/kg/day) and blood transfusion. Moreover, granulation tissue was raised with a fixation of NPWT (Fig. 2h). For wound closure, the wounds in perineum, right buttock and partial abdomen were sutured, while skin graft was used to cover the residual defect in abdomen (Fig. 2i, j). The patient was discharged on the 29th day after admission and no relapse was observed.

## Discussion

The term of FG refers to necrotizing fasciitis that involves perineum and perianal region due to the pathognomonic features. Existing studies have revealed a predominance among the elderly with a mean age of over 50 years, which coincides with this paper. Martinez found that old age was not a direct factor affecting survival [5]. However, this aging trend seems to be related to the incidence of FG with an increasing number of predisposing disease. Similar to our results, males are more common to suffer from FG than females, with the reported male-to-female ratio of 5.3:1 [6]. This sex distribution appears to be associated with the anatomical difference. Infection spreads along the fascial plane, but due to the anatomic protection of the envelope of urethra and corpora cavernosa, scrotum is more common to be affected. It is considered that females are less susceptible to this infection, because of the better drainage of secretion in the female pelvis. Testicular necrosis is rare as a result of the independent blood supply of internal spermatic artery. Therefore, if an infection involves the testis, it should be wary of intraperitoneal or retroperitoneal infections [7]. However, in the case of females, it may cause severe extension to the anterior abdominal wall, as Colles’ fascia is not anteriorly attached.

The underlying pathogenesis of FG is the spread of local infection in the broad sense, only 10% cases are considered idiopathic [8]. The bacteria isolated from FG wound are consistent with normal flora in the urogenital and anogenital regions, which include Gram-negative, Gram-positive pathogens and anaerobic bacteria, although anaerobes are less frequently isolated [9]. As reported in studies, the most susceptible bacteria are *Escherichia coli*, *Pseudomonas aeruginosa*, *Enterococcus faecium* and *Staphylococcus aureus* [10]. Besides, FG presents a combination of mixed bacteria infection, which may be related to the rapid disease progression and severity. Such complex polymicrobial infection is another feature of FG compared with necrotizing fasciitis in other area. So broad-spectrum antibiotics should be chosen as the basic antibacterial therapy. Generally, double- or triple-combination is recommended as the basic criterion, including third-generation cephalosporins, aminoglycoside antibiotics, metronidazole or ornidazole [5]. Furthermore, new drugs, such as meropenem and piperacillin-tazobactam, are also advocated for the sake of larger distribution and less renal toxicity. In early admission, the regimens are applied empirically in this series, but antibiotics must be modified as appropriate according to the culture results.

The predisposing disease is not only important in the occurrence of FG, but also in its mortality. Among these predisposing diseases, DM is identified as the most common comorbidity involved in FG, which may increase the possibility of bacterial infection. It is found in the literature review that, the percentage of DM reaches up to 76.9% in patients with FG [11]. Moreover, the degree of DM control has also been demonstrated to be correlated with disease progression and extent, therefore, patients with uncontrolled DM may be associated with dismal prognosis [12]. It should be noted that, DM control is difficult in the early progression of FG because of the severe inflammatory response and immune disorder. As discovered from the clinical observation in this study and previous reports, insulin pump is more convenient and efficient than normal insulin injection for the unruly blood glucose [13]. Other predisposing diseases include abscess in perineum and perianal region, hypertension, local trauma, chronic catheterization, tumor, and organ failure. In a sense, Chen considered FG as a complication of colorectal or urological diseases [14]. While, among these predisposing diseases, organ failure should be paid special attention, especially for renal failure. Because deteriorated renal function can not only greatly increase the incidence of FG, but also have an impact on mortality. Moreover, multiple predisposing diseases are a direct indicator for the prediction of mortality [15].

Early diagnosis plays a crucial role in the management of FG, but the diagnosis of FG is particularly challenging for clinicians in two aspects. On the one hand, early clinical symptoms, including erythema and swelling, can be confused with other infections, such as abscess, fistula or local trauma. Besides, skin necrosis due to vascular thrombosis often falls behind the progression of fascial infection. As demonstrated by Chawla, the necrotic area showed no direct relationship with the disease severity or prognosis [16]. Therefore, it is difficult to make a definite diagnosis in the absence of pathognomonic manifestation at the early stage. On the other hand, delayed diagnosis is fatal, which is correlated with a much higher mortality rate, since the infection progression rate can reach up to 2–3 cm/h. Particularly, septicemia can occur within an hour after the disease outbreak.

In this series, early diagnosis depends on the combination of clinical symptoms, imaging findings and laboratory tests. Apart from the typical symptoms of infection, crepitus caused by gas bubbles can also be found within the infected area as a special feature, and gas in scrotal wall can be detected on ultrasound even before the presence of clinical crepitus. In a literature review, about 54.3% cases are complicated with the symptom of crepitus [17]. The gas on the fascial plane shows dirty shadowing and discrete hyperechoic focus in ultrasonography. While it should be noted that the absence of soft tissue gas does not exclude FG. Other laboratory tests, including acidosis, leukocytosis, sudden thrombocytopenia, and anemia, may be helpful for the early diagnosis. The LRINEC score, which is a combination of indicators, is more practical, and a score ≥ 8 is strongly predictive of FG. Nonetheless, it is demonstrated that LRINEC is only helpful for diagnosis, but fails to predict the clinical outcome. Moreover, the LRINEC score is more sensitive for patients with electrolyte variation and renal impairment [18].

Aggressive surgical treatment is advocated for the highly suspected cases. A definite diagnosis can be made based on the intraoperative manifestation that invasive infection is observed to spread along the deep fascia. Surgical debridement is conducted to remove the necrotic tissue and make adequate drainage. Typically, the debridement boundary should reach the normal fascia, rather than the normal skin. The following repeated surgical interventions are necessary in the case of infection progression or abundant necrotic tissue. In the meantime, worries about the relationship with mortality may also exist. According to an analysis of 19 FG patients, the average number of repeated debridement was 3.5 times/case, which appeared to have no impact on the patient outcome [16]. It is also demonstrated that, those who are destined not to survive cannot tolerate the repeated debridement necessary for survival.

As for wound management, granulation tissue is cultured for further reconstructive treatment. Typically, NPWT is the most commonly applied method, which is advantageous in wound healing with physiological effects [19]. The superiorities of NPWT have been approved by several studies, yet there are still shortcomings, including high expense, fixed difficulties and skin irritation. Further, NPWT is fittable for cases receiving colostomy. In the absence of fecal diversion, NPWT often leaks due to the fecal excretion. In this series, NPWT was applied in the wound located in scrotum, penis, abdomen, thigh, and perianal region with colostomy. For the perianal cases without ostomy, normal dressing changes were adopted. However, the necessity of fecal diversion is still controversial at present. On the one hand, fecal diversion changes the excretion pathway and facilitates nutrition support; on the other hand, it remains unclear whether it can reduce the risk of infection [2]. In our opinion, the decision of fecal diversion should be made after taking patient conditions and disease progression into full consideration. There is no need to perform forced ostomy for the convenience of NPWT.

Diverse ways can be selected for wound closure, including secondary healing or suture, flaps and skin grafts. But a primary concern is focused on the closure of scrotal defect, given the long-term function of testicle and spermatogenesis. Skin graft is denounced for contraction and abrasion, regardless of its simple procedure and fewer complications. At the same time, the flap derived from the disruption of scrotal thermoregulation with thick flap or thigh testicular transposition is also a source of concern [20]. At present, robust evidence and long-term observation are warranted to conclude an ideal way for scrotal defect closure. An advisable choice of reconstructive procedure should be made based on the individual characteristics, patient preference and surgeon experience. As shown in Table 1, for small scrotal defect (less than 50%), advancement flap or secondary suture was adopted because the scrotal skin was elastic and stretchable. By contrast, the split-thickness skin graft was adopted for larger defect, and pudendal-thigh flap was used in the presence of deep dead space, so as to eliminate the dead space in scrotum.

The mortality in this paper seemed to be much lower than that in previous report. However, this mortality rate was underestimated, since the abandoned cases were excluded according to our inclusion criteria, while they were suspected with a high probability of death. Up to the present, the mortality is considered to be stable, since no obvious evolution is attained in the existing treatment measures for infection control. It is advisable to make efforts to prevent and diagnose FG early. Moreover, the multidisciplinary cooperation model should be adopted in future treatment, including anorectum surgery, urology, infection department and critical care medicine.

Obviously, the shortage of this study is the limited cases, which is much attributed to the lower incidence rate of FG compared to necrotizing fasciitis in other regions. While our aim is to summarize the treatment experience, and make improvement for further treatment. Moreover, adjunctive hyperbaric oxygen was not applied in this study. Although it was reported with a decrease of mortality rate in FG patients after hyperbaric oxygen therapy, the studies were mainly case reports. Due to the shortage of randomized controlled trials and robust theoretical support, further application of hyperbaric oxygen therapy is still limited [21].

## Conclusion

In conclusion, FG is a life-threatening infection that requires early diagnosis and debridement. The predisposing disease, clinical manifestation and LRINEC score should be taken into comprehensive consideration, which is helpful for timely diagnosis. Moreover, further successful treatment depends on the aggressive surgery intervention, broad-spectrum antibiotics therapy, wound management and closure choice.

## Data Availability

The datasets used during the current study are available from the corresponding author on reasonable request.

## Abbreviations

FG: Fournier’s gangrene
WBC: White blood cell
DIC: Disseminated intravascular coagulation
MOF: Multiple organ failure
LRINEC: Laboratory risk indicator for necrotizing fasciitis
DM: Diabetes mellitus
BP: Blood pressure
HR: Heart rate
CRRT: Continuous renal replacement therapy
NPWT: Negative pressure wound therapy
MRSA: Methicillin-resistant *Staphylococcus aureus*
